# Characterization of meibomian gland dysfunction in patients with rosacea

**DOI:** 10.5935/0004-2749.20230043

**Published:** 2023

**Authors:** Eduardo Buzolin Barbosa, Carla Melo Tavares, Dimitri Felipe Luz da Silva, Lorraine Souza Santos, Andrea Fernandes Eloy da Costa França, Monica Alves

**Affiliations:** 1 Department of Ophthalmology and Otorhinolaryngology, Faculdade de Ciências Médicas, Universidade Estadual de Campinas, Campinas, SP, Brazil.; 2 Department of Internal Medicine, Division of Dermatology, Faculdade de Ciências Médicas, Universidade Estadual de Campinas, Campinas, SP, Brazil.

**Keywords:** Rosacea/complications, Meibomian gland dysfunction, Conjunctiva, Dry eye syndromes, Diagnostic techniques, ophthalmological, Rosácea/complicações, Disfunção da glândula tarsal, Túnica conjuntiva, Síndromes do olho seco, Técnicas de diagnóstico oftalmológico

## Abstract

**Purpose:**

To compare ocular surface parameters in rosacea patients with those of controls.

**Methods:**

Ninety-three participants took part in this cross-sectional, observational, non-interventional study. These consisted of a rosacea group (n=40) and a control group (n=53). We compared objective parameters of the ocular surface, including conjunctival hyperemia, tear film stability and volume, meibomian gland dysfunction, dry eye disease, and ocular surface staining, between the two groups.

**Results:**

In the rosacea group, 69.23% were female. The mean age was 47.34 ± 12.62 years old. No statistically significant differences between groups were found in visual acuity (p=0.987), tear film parameters (tear meniscus height (p=0.338), noninvasive tear film rupture time (p=0.228), invasive rupture time (p=0.471), Schirmer’s test scores (p=0.244), conjunctival hyperemia (p=0.106), and fluorescein staining (p=0.489). Significant differences were found in meibography evaluations (p=0.026), mucous layer integrity (p=0.015), and ocular surface symptoms (p<0.0001). Rosacea patients also showed important eyelid differences in glandular expressibility (p<0.001), glandular secretion pattern (p<0.001), and telangiectasia (p<0.001) compared to controls.

**Conclusion:**

Meibomian gland dysfunction is frequently associated with dermatological conditions. It can be observed in morphological findings from meibography as well as lipid secretion impairment, leading to evaporative dry eye, ocular surface dysfunction, and inflammation.

## Abstract (pt)

**Objetivo:**

Avaliar as alterações da superfície ocular em pacientes com Rosácea, e comparar com grupo controle.

**Métodos:**

Noventa e três indivíduos foram selecionados para este estudo transversal, observacional e não intervencionista, dividido em dois grupos: rosácea (n=40) e controles (n=53). Foram avaliados parâmetros objetivos da superfície ocular (hiperemia conjuntival, estabilidade e volume do filme lacrimal, disfunção da glândula meibomiana, doença do olho seco, coloração da superfície ocular) e comparado indivíduos saudáveis com pacientes com rosácea.

**Resultados:**

69,23% dos indivíduos com rosácea eram mulheres, com média de idade de 47,34 ± 12,62 anos. Em comparação com controles pareados, não foram evidenciadas diferenças estatisticamente significativas em relação à acuidade visual (p=0,987) e parâmetros do filme lacrimal (altura do menisco lacrimal (p=0,338), tempo de ruptura do filme lacrimal não invasivo (p=0,228), tempo invasivo de ruptura (p=0,471) e teste de Schirmer (p=0,244), bem como hiperemia conjuntival (p=0,106) e coloração com fluoresceína (p=0,489). Associação significativa foi encontrada na avaliação da meibografia (p=0,026), integridade da camada mucosa (p=0,015) e sintomas de superfície ocular (p<0,0001). Pacientes com rosácea também apresentaram alterações importantes na borda palpebral: expressibilidade glandular (p<0,001), padrão de secreção glandular (p<0,001) e telangiectasia (p<0,001).

**Conclusão:**

A disfunção da glândula de Meibômio está frequentemente associada a condições dermatológicas e é caracterizada por achados morfológicos na meibografia, bem como comprometimento da secreção lipídica que leva ao olho seco evaporativo e alterações da superfície ocular e inflamação.

## INTRODUCTION

Rosacea is a chronic inflammatory skin condition characterized by major and secondary cutaneous symptoms that include flushing, telangiectasia, papules, pustules, and ocular abnormalities. Multiple features may occur in the same patient but the standard classification system of the National Rosacea Society Expert Committee (2002) is still used for didactic purposes. This classifies the disorder into four subtypes: erythematotelangiectatic, papulopustular, phymatous, and ocular rosacea^(1-3)^.

Ocular involvement is common but is often overlooked in patients with rosacea^(4)^. Around 58 to 72% of rosacea patients experience ocular symptoms. These are usually mild and nonspecific^(4,5)^. When cutaneous symptoms are unremarkable, ocular rosacea may be misdiagnosed as an eye condition^(4)^. Patients can experience ocular burning, itching, redness, photophobia, and foreign body sensations^(6,7)^. Objective signs of ocular rosacea are lid margin telangiectasia, interpalpebral conjunctival injection, spade-shaped infiltrates in the cornea, and scleritis or sclerokeratitis^(8)^. Lid disease-related manifestations such as blepharitis and meibomian gland dysfunction are the most common presentations, but abnormal Schirmer test findings and corneal involvement have been reported in more than one-third of cases^(9)^. Less specific findings such as conjunctivitis, collarettes around the lashes, abnormal meibomian secretion, and evaporative tear dysfunction also seem to be common but poorly detailed^(4,5,9)^. In this context, ocular surface conditions related to rosacea remain poorly described.

This study aims to evaluate ocular surface findings in rosacea patients, quantifying symptoms and measuring objective ocular surface parameters. Correlations between ocular manifestations and cutaneous disease presentation will provide a better understanding of the full disease spectrum and may help both ophthalmologists and dermatologists to provide the most appropriate treatment for this complex disease.

## METHODS

This was a cross-sectional, observational, non-interventional study. Forty rosacea patients were included along with a control group of 53 healthy individuals. Participants were matched between groups by age and sex. Patients were recruited from dermatology and ophthalmology outpatient clinics at the University of Campinas (UNICAMP) between 2017 and 2019 and for control group age and sex matched participants were recruited from hospital staff and non ocular surface disease patients. Individuals with other ocular surface diseases, such as sequelae of trachoma and herpetic keratitis, and with other dry eye conditions, such as Sjogren’s syndrome, were excluded. This study was carried out with the approval of the Institutional Research Ethics Committee Board of the University of Campinas (UNICAMP). Written informed consent was obtained from all subjects before any procedures were performed.

Classification and rosacea staging were based on the 2002 report of the National Rosacea Society Expert Committee^(2,8)^ and the Dermatological Life Quality Index (DLQI)^(10,11)^ and were performed by a dermatologist. All participants underwent a detailed ophthalmological examination. The tests and measures were as described below and were performed in the sequence given.

After a comprehensive ocular anamnesis, dry eye symptoms were evaluated using the ocular surface disease index (OSDI) questionnaire. OSDI scores range from 0 to 100, with values below 12 considered normal^(12,13)^.

The ocular surface parameters analyzed were as follows:

- Tear meniscus height (TMH): Tear film volume;
- Noninvasive tear breakup time (NITBUT): Tear film stability;
- Meibography: Meibomian gland morphology;
- Fluorescein staining: corneal epithelial integrity;
- Lissamine green staining: Damage to ocular surface epithelial cells and absence of mucin or glycocalyx protection;
- Schirmer test: Tear volume.

Measures of TMH and NITBUT, and meibography were obtained using the Keratograph 5M (Oculus; Wetzlar, Germany), a noninvasive device developed for objective assessment and photographic documentation of the tear film and ocular surface. All procedures were sequentially performed by the same examiner in accordance with specific guidelines and regulations^(13-16)^.

Ocular surface disease was classified according to the global consensus of the Tear Film and Ocular Surface Society Dry Eye Workshop II (TFOS DEWS II) and the International Workshop on Meibomian Gland Dysfunction. Table 1 summarizes the parameters and cutoff values for discrimination between the two main subtypes of dry eye. These are aqueous deficient (low tear volume) and evaporative dry eye (lipid deficient). Patients with OSDI scores ≥13 and noninvasive tear film breakup time <10 s or corneal staining >5 spots or conjunctival staining >3 were diagnosed with dry eye. Of these dry eye patients, those with a tear meniscus height £0.2 mm were classified as having the aqueous tear deficiency subtype, and those with a meiboscore grade ≥1 were classified as having meibomian gland dysfunction and evaporative dry eye. Patients who met both criteria were classified as having mixed type dry eye^(13,16,17)^.

**Table 1:** Classification of dry eye disease

Dry eye classification	Criteria
**Dry eye disease**	OSDI score ≥13 AND Noninvasive tear film breakup time <10 s, corneal staining >5 spots, conjunctival staining >3 (10-15)
**Aqueous tear deficiency**	Diagnosis of dry eye disease AND Tear meniscus height £0.2 mm
**Meibomian gland dysfunction**	Diagnosis of dry eye disease AND Meibography grade ≥ 1
**Mixed dry eye**	Dry eye disease in the presence of aqueous tear deficiency and meibomian gland dysfunction

ODSI= Ocular surface disease index.

### Statistical analyses

Exploratory data analysis was performed using descriptive statistics (mean, standard deviation, median, range, frequency, and percentage). Multiple logistic regression was used to assess factors associated with the most frequent types of rosacea. The significance level was set at p£0.05. Statistical analyses were performed using STATA 14.0 software (StataCorp LP; College Station, TX, USA).

### Ethics statement

This study was approved by the Institutional Research Ethics Committee Board of the University of Campinas (UNICAMP) (approval number: 80618117.0.0000.5404). It was conducted in accordance with the guidelines of the Declaration of Helsinki (1964). Written informed consent was obtained from all subjects before any procedures were performed.

## RESULTS

Detailed demographic and clinical patient data are presented in table 2. The majority of rosacea participants were female (69.23%) and the mean age was 47 years (range = 23 to 75). In the matched control group, 66% were female and the mean age was 44 years. Patients were categorized into the four rosacea subtypes: erythematotelangiectatic, papulopustular, phymatous, and ocular rosacea. Those that qualified for more than one subtype of rosacea were classified as mixed type. Erythematotelangiectatic was the most common (49%) subtype. Only three patients (7.5%) had a previous diagnosis of ocular rosacea and one had exclusive ophthalmological involvement. Rosacea severity was mild in the majority of patients (52.5%). The DLQI found rosacea to have no, or minimal, impact on patients’ life (DLQI ≤5) in 62.5% of cases, with the highest values related to more severe cutaneous symptoms (p=0.018). Although 62.5% of the patients had dry eye symptoms according to their OSDI scores, most had never sought eye treatment.

**Table 2:** Clinical and demographic features of the rosacea group in our sample

Variable	N=40	Frequency (%)
**Age (Mean ± SD)**	47 ± 12	
Sex (M/F)	12/28	30%/ 70%
Fitzpatrick scale		
1 and 2	25	62.5%
3 and over	15	37.5%
Rosacea subtype*		
Erythematotelangiectatic	25	
Papulopustular	18	
Other (phymatous, ocular, mixed)	08	
DLQI		
0 to 5 (no or small effect)	25	62.5%
6 to 10 (moderate effect)	10	25%
>10 (large effect)	5	12.5%
Global assessment		
Absent/mild	23	57.5%
Moderate/severe	17	42.5%
Treatment		
No treatment	6	15%
Topical	16	40%
Systemic	14	35%
Other	4	10%

SD= Standard deviation; DLQI= Dermatological Life Quality Index; M= Male; F= Female.

* Patients could present with more than one subtype of rosacea.

Forty patients diagnosed with rosacea and 53 healthy controls, matched by age and sex, were evaluated. Table 3 shows the findings from our measures of ocular parameters for each group. Rosacea patients had higher OSDI scores, greater meibomian gland dysfunction (identified in meibography evaluation), and greater mucin layer involvement (measured by lissamine green staining) than the control group.

**Table 3:** Ocular surface parameters of rosacea patients and controls

Parameter	Control	Rosacea	*P*-value
	Mean ± SD (Median)	Mean ± SD (Median)	
OSDI	6.01 ± 9.40 (2.10)	26.30 ± 22.10 (20.83)	<0.0001*
Tear meniscus (mm)	0.24 ± 0.06 (0.23)	0.22 ± 0.07 (0.22)	0.3382
NITBUT (seconds)	8.83 ± 5.26 (7.26)	7.81 ± 5.40 (5.93)	0.2282
Conjunctival redness (grade 0-4)	1.23 ± 0.64 (1.20)	1.46 ± 0.61 (1.30)	0.1064
Meibography	0-17.30% (9)	0-11.76% (4)	
	1-67.30% (35)	1-52.94% (18)	
	2-15.38% (8)	2-26.47% (9)	
	3-0% (0)	3-11.76% (4)	**0.0258** *
Fluorescein staining (grade 0-15)	0.47 ± 0.64 (0.00)	0.74 ± 1.07 (0.00)	0.4887
Invasive TBUT (seconds)	8.02 ± 4.48 (7.00)	6.97 ± 2.85 (7.00)	0.4709
Lissamine staining (grade 0-9)	0.90 ± 1.27 (0.00)	1.51 ± 1.43 (1.00)	0.0152*
Schirmer’s test (mm)	15.61 ± 11.35 (15.00)	14.03 ± 13.46 (9.50)	0.2438

ODSI= ocular surface disease index; NITBUT= noninvasive tear breakup time; TBUT= tear breakup time; SD= standard deviation;

* *p*<0.05 (Mann-Whitney U-test).

The Tear Film and Ocular Surface Society (TFOS) Dry Eye Workshop II (DEWS II)^(13,16,18-21)^ and the International Workshop on Meibomian Gland Dysfunction^(17,22-24)^ have found that almost half of rosacea patients (41%) meet the criteria for dry eye. In our sample, 62.5% of the rosacea group were found to have evaporative dry eye, 6.25% had aqueous deficiency, and 31.25% had mixed type dry eye.

**Figure 1:**
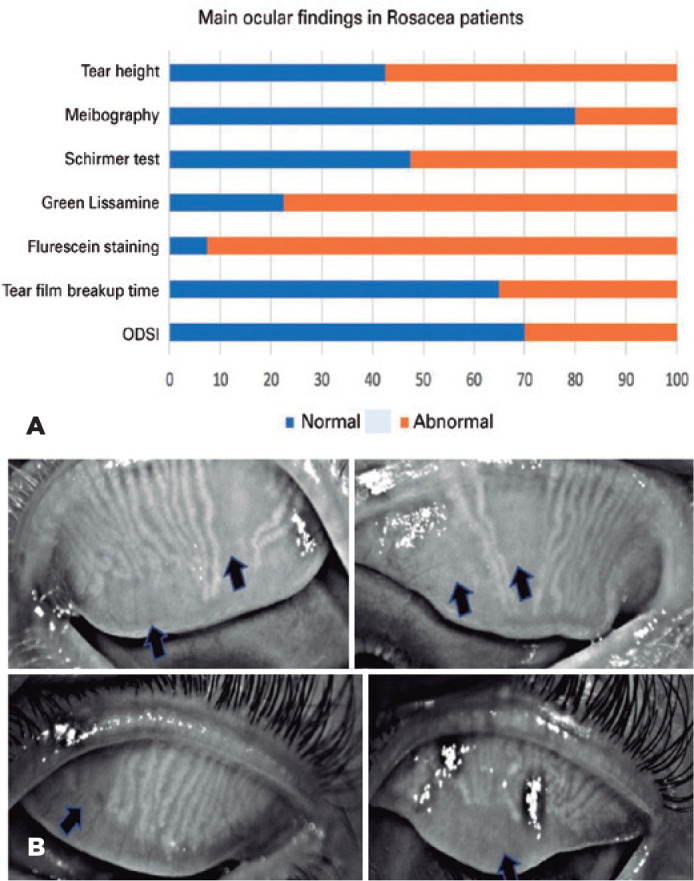
Main ocular findings in rosacea patients. (A) Frequency of ocular parameters (in percentage). OSDI: Ocular Surface Disease Index questionnaire. (B) Meibomian Gland Dysfunctions in Rosacea patients. Arrows showing glandular dropout.

**Figure 2:**
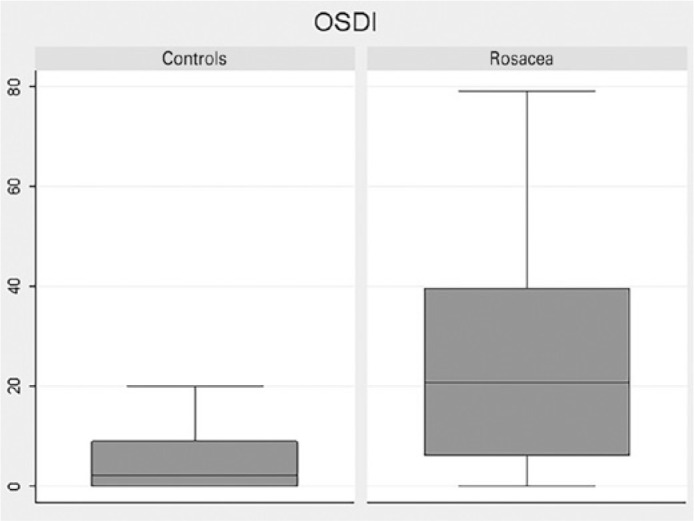
Blot spot comparison of ocular surface disease index (OSDI) scores of the control group and the rosacea group. Values below 12 are considered normal.

All of the rosacea patients in this study showed some degree of meibomian gland dysfunction. Glandular morphology and eyelid evaluations were performed. Higher meiboscores, telangiectasia, and pasty glandular secretions were the most frequent findings (Table 3). Figure 1 displays the ratios of abnormal to normal findings for each ocular parameter evaluated, with abnormalities ranging from 22% to more than 80%. Table 4 provides a comparison of ocular parameter findings between rosacea subtypes. Figure 2 displays the differences in OSDI scores between the control group and the rosacea group.

**Table 4:** Ocular and eyelid border parameters of rosacea subtypes

Parameters	Erythematous	Papulopustular	p-value
Symptoms			
OSDI	39.76 ± 24.55 (31.25)	18.52 ± 15.77 (20.83)	0.0370*
Tear volume			
Tear meniscus height	0.22 ± 0.06 (0.21)	0.23 ± 0.09 (0.23)	0.7252
Schirmer’s test	11.62 ± 13.68 (6.50)	15.77 ± 12.01 (12.0)	0.2104
Tear stability			
NITBUT	7.82 ± 5.18 (6.69)	9.03 ± 6.76 (5.90)	0.7923
Invasive TBUT	7.31 ± 3.18 (7.0)	6.77 ± 2.59 (7.0)	0.5959
Inflammation			
Conjunctival redness	1.36 ± 0.49 (1.30)	1.38 ± 0.41 (1.40)	0.8603
Ocular surface damage			
Fluorescein staining	0.81 ± 1.27 (0)	0.54 ± 0.78 (0)	0.8006
Lissamine staining	1.87 ± 1.82 (1.0)	1.31 ± 1.18 (1)	0.5858
Meibomian gland dysfunction			
Meibography	0-11.76% (2)	0-15.38% (2)	0.2145
	1-47.05% (8)	1-46.15% (6)	
	2-23.52% (4)	2-30.76% (4)	
	3-17.64% (3)	3-7.69% (1)	
Normal secretion			p=1.000
Yes	0	0	
No	100%	100%	
Obstructed glands			p=0.227
Yes	57.14%	27.27%	
No	42.86%	72.73%	
Granular secretion			p=0.697
Yes	35.71%	45.45%	
No	64.29%	54.55%	
Pasty secretion			
Yes	14.29%	54.55%	**p=0.043** ^ø^
No	85.71%	45.45%	
Telangiectasias			p=0.209
Yes	85.71%	63.64%	
No	14.29%	36.36%	

OSDI= Ocular surface disease index; NITBUT= Noninvasive tear breakup time; TBUT= Tear breakup time.

Continuous data are expressed as mean ± standard deviation (median). Categorical variables are expressed as frequency (%).

* *p*<0.05 (Mann-Whitney U-test);

ø *p*<0.05 (Fisher’s exact test).

Additionally, detailed analyses of our ophthalmological findings comparing different global evaluations of rosacea and different treatments were performed but no significant associations were found.

## DISCUSSION

Ocular symptoms are the most common extracutaneous manifestation of rosacea. These symptoms may even precede cutaneous involvement, affecting the ocular surface and meibomian glands, leading to dry eye disease^(4,5)^.

Recent research has identified the tendency to dry eye in patients with rosacea through their lower Schirmer test results, shorter tear film breakup time, and higher OSDI scores than non-rosacea individuals^(4,5,7,25,26)^.

In this cross-sectional cohort of rosacea patients, ocular surface disease symptoms and meibomian gland dysfunction were frequent findings. Dry eye diagnosis encompasses A broad range of tests are used for dry eye diagnosis to create a precise picture of individual variations in tear film and ocular surface parameters. In this study, dry eye was diagnosed in patients with an OSDI >13 and positive results on one clinical test. The dry eye was classified as evaporative when it was related to meibomian gland dysfunction and as aqueous deficient when it was secondary to diminished tear production. When both symptoms were present, it was classified as mixed type dry eye. Thereby, this evaluation and classification provided a comprehensive account of ocular surface disease in rosacea patients. We found evidence for all ocular surface disease parameters in this group of patients, ranging from 22% to more than 80%. Most commonly, the rosacea patients were found to have meibomian gland dysfunction, higher symptom scores on the OSDI, and positive lissamine green staining. The latter is indicative of damage to corneal and conjunctival cells. Of note, was a high frequency of glandular abnormality, observed in expressibility and secretion pattern evaluation of the eyelid margins. Rosacea was associated with ductal obstruction, telangiectasia, and altered glandular secretion, with patterns of granular or pasty secretion. All dry eye subtypes were found^(13,16)^.

Analysis was conducted of variable differences between the rosacea subtypes: erythematotelangiectatic, papulopustular, phymatous, and ocular. Multiple logistic regression showed the erythematotelangiectatic subtype to have the worst OSDI scores. However, most of the patients with papulopustular rosacea were receiving systemic antibiotic treatment, which also treats ocular manifestations. This finding reinforces the need to look for ocular symptoms in all individuals with rosacea, regardless of the clinical form, stage, or global assessment of the disease. Rosacea patients should routinely receive complete ocular evaluations.

Our study highlights the importance of searching for ocular symptoms in rosacea. OSDI is a noninvasive means of assessing ocular surface disease. It is a quick, easily administered questionnaire that can be used by dermatologists to identify rosacea patients who require further ophthalmological evaluation.

The cross-sectional, single-center design was a limitation of this study. Another limitation was our failure to investigate possible demodex infection since rosacea patients have a higher prevalence of demodex infestation. Demodex mites can be found in the eyelashes of normal populations, with the rate and density of mite infestation increasing with age. However, the degree of demodex infestation seems to play an important role in the inflammation process of rosacea. Demodex infestation is associated with bacterial load and mite allergens. In patients with rosacea, these can further aggravate the abnormal immune response and the development of ocular surface disease(^27,28^). The main strength of this study is the systematic ocular evaluation, using a broad panel of tests, to generate a comprehensive characterization of all ocular surface parameters in rosacea and find correlations between these parameters and the clinical presentation of this complex disease.

Literature is scarce on the relationship between rosacea and ocular surface disease, and, to our knowledge, this study is one of the largest to perform complete and systematic ocular assessments of rosacea patients. Our results reinforce the findings of Palamar et al.^(29)^ and Machalińska et al.^(30)^ regarding the associations between rosacea and meibomian gland dysfunction, eyelid abnormalities, and dry eye disease. Those conditions may share pathological mechanisms and potential therapeutic responses.

Severe forms of ocular surface disease, such as corneal complications secondary to dry eye and inflammation, have significant consequences for patients. Improved understanding of the ocular manifestations of rosacea disease will enable both dermatologists and ophthalmologists to provide better care and treatment to affected patients. The quantification of symptoms and identification of meibomian gland dysfunctions as a prevalent feature in rosacea should both be pursued further. Our ocular findings may be utilized as clinical tools in the screening and follow-up of this condition to guarantee ocular surface integrity and prevent complications.
